# Ovarian Function Modulates the Effects of Long-Chain Polyunsaturated Fatty Acids on the Mouse Cerebral Cortex

**DOI:** 10.3389/fncel.2018.00103

**Published:** 2018-04-24

**Authors:** Jose L. Herrera, Lara Ordoñez-Gutierrez, Gemma Fabrias, Josefina Casas, Araceli Morales, Guadalberto Hernandez, Nieves G. Acosta, Covadonga Rodriguez, Luis Prieto-Valiente, Luis M. Garcia-Segura, Rafael Alonso, Francisco G. Wandosell

**Affiliations:** ^1^Departamento de Ciencias Médicas Básica and Instituto de Tecnologías Biomédicas, Centro de Investigaciones Biomédicas de Canarias, Universidad de La Laguna, La Laguna, Spain; ^2^Centro de Biología Molecular “Severo Ochoa” (CSIC-UAM), Universidad Autónoma de Madrid, Madrid, Spain; ^3^Centro de Investigación Biomédica en Red de Enfermedades Neurodegenerativas, Madrid, Spain; ^4^Instituto de Química Avanzada de Cataluña (IQAC-CSIC), Barcelona, Spain; ^5^Departamento de Biología Animal, Edafología y Geología, and Instituto de Tecnologías Biomédicas, Centro de Investigaciones Biomédicas de Canarias, Universidad de La Laguna, Tenerife, Spain; ^6^Unidad de Estadística Médica, Universidad Católica de Murcia, Murcia, Spain; ^7^Instituto Cajal (CSIC) and Centro de Investigación Biomédica en Red de Fragilidad y Envejecimiento Saludable, Madrid, Spain

**Keywords:** cerebral cortex lipidome, long-chain polyunsaturated fatty acids (LC-PUFAs), docosahexaenoic acid (DHA), sphingolipids, ovarian hormones, synaptic proteins

## Abstract

Different dietary ratios of *n*−6/*n*−3 long-chain polyunsaturated fatty acids (LC-PUFAs) may alter brain lipid profile, neural activity, and brain cognitive function. To determine whether ovarian hormones influence the effect of diet on the brain, ovariectomized and sham-operated mice continuously treated with placebo or estradiol were fed for 3 months with diets containing low or high *n*−6/*n*−3 LC-PUFA ratios. The fatty acid (FA) profile and expression of key neuronal proteins were analyzed in the cerebral cortex, with intact female mice on standard diet serving as internal controls of brain lipidome composition. Diets containing different concentrations of LC-PUFAs greatly modified total FAs, sphingolipids, and gangliosides in the cerebral cortex. Some of these changes were dependent on ovarian hormones, as they were not detected in ovariectomized animals, and in the case of complex lipids, the effect of ovariectomy was partially or totally reversed by continuous administration of estradiol. However, even though differential dietary LC-PUFA content modified the expression of neuronal proteins such as synapsin and its phosphorylation level, PSD-95, amyloid precursor protein (APP), or glial proteins such as glial fibrillary acidic protein (GFAP), an effect also dependent on the presence of the ovary, chronic estradiol treatment was unable to revert the dietary effects on brain cortex synaptic proteins. These results suggest that, in addition to stable estradiol levels, other ovarian hormones such as progesterone and/or cyclic ovarian secretory activity could play a physiological role in the modulation of dietary LC-PUFAs on the cerebral cortex, which may have clinical implications for post-menopausal women on diets enriched with different proportions of *n*−3 and *n*−6 LC-PUFAs.

## Introduction

Phospholipids are major components of neural cell membranes, playing critical roles in synaptic transmission and neuronal signaling through interactions with specific membrane proteins (Bazan, 2014). The brain's complement of phospholipids and other complex lipids contains large amounts of long-chain polyunsaturated fatty acids (LC-PUFAs) such as arachidonic acid (ARA; 20:4*n*−6) and docosahexaenoic acid (DHA; 22:6*n*−3), but low levels of other omega-3 LC-PUFAs, especially eicosapentaenoic acid (EPA; 20:5*n*−3) (Brenna and Diau, 2007; Chen et al., 2009). While saturated and monounsaturated fatty acids can be synthesized *de novo* within the brain, LC-PUFAs are mainly supplied by the blood and are synthesized from two dietary precursors: linoleic acid (LNA; 18:2*n*−6) and α-linoleic acid (ALA; 18:3*n*−3) (Carrié et al., 2000; Williard et al., 2001; Bazinet and Layé, 2014). Even though the specific mechanisms involved in phospholipid signaling are not completely understood, several pro-resolving lipid mediators (SPMs) have been identified, including 17S-hydroxy-DHA (17S-DHA), neuroprotectin D1 (NPD-1), resolvin D5 (RvD5), 14S-HDHA and maresin 1 (MaR1) (Orr et al., 2013; Serhan, 2014). LC-PUFAs and SPMs participate in a plethora of signaling processes, such as cell survival and neuroinflammation, neurotransmission, and cognitive function (Bannenberg and Serhan, 2010; Serhan and Chang, 2013). One of the best-characterized SPMs is NPD-1, which is synthesized in response to brain injury and may have therapeutic potential in a wide range of neurological conditions (Bazan et al., 2011a,b, 2013).

In rodents, daily intake of omega-3 LC-PUFAs (*n*−3 LC-PUFAs) is necessary for neural development and maintenance of synaptic circuitry (Brenna and Diau, 2007; Dyall and Michael-Titus, 2008; Bazan et al., 2011a,b). In addition, experimental and clinical data support that their dietary inclusion has positive effects on numerous pathological conditions (Gorjão et al., 2009; Mozaffarian and Wu, 2012; Russell and Bürgin–Maunder, 2012). Diets enriched in *n*−3 LC-PUFAs have been associated with a lower incidence of dementia and neurological disorders (Mazza et al., 2007), while diets containing low percentage of DHA may be linked to cognitive impairment (Ikemoto et al., 2001; Catalan et al., 2002). Interestingly, the ratio of *n*−3 to *n*−6 LC- PUFA in the diet appears to be a critical factor for the effect on the brain lipidome (Bourre et al., 1984; Jumpsen et al., 1997; Carrié et al., 2000). On the other hand, women have significantly lower blood levels of docosapentaenoic acid (DPA; 22:5-3) and EPA, but significantly higher levels of DHA than men (Metherel et al., 2009). In studies with female mice, it has been reported that the effect of diet on the brain lipidome may be partially dependent on circulating levels of gonadal hormones (Díaz et al., 2016). Furthermore, experimental evidence has indicated that estrogen may modulate neuronal signaling through interactions with specific estrogen receptors in neural membrane microdomains, such as lipid rafts (Herrera et al., 2011; Marín et al., 2013).

The aim of this study was to investigate the putative synergistic interaction between estradiol and differential dietary fatty acid (FA) supplementation, and observe how this combination may affect the long-term lipid and protein profile in the brain. A multifactorial design was established to investigate the effects of diets containing two different ratios of *n*−3 and *n*−6 LC-PUFAs, specifically EPA and DHA, and their potential interaction with reproductive status in placebo and estradiol-treated ovariectomized mice. At the end point, analysis of the cerebral cortex showed that both diets modified the brain lipidome when compared to standard diet, and may influence neural function by modifying the content of several proteins involved in intracellular signaling and synaptic transmission. In addition, some of these effects were sensitive to the presence or absence of the ovaries and, at least partially, to circulating levels of estradiol.

## Materials and methods

### Animals and husbandry

Female C57BL/6J mice (*Mus musculus*) were purchased from Charles River Laboratories, and housed under constant temperature (22 ± 2°C) and humidity (50 ± 5%) and a 12:12 h light-dark cycle in specific pathoge*n*–free conditions. Mice (10 per cage) were housed in cages fitted with microbarrier filter tops, and allowed access to food (A03/R03, SAFE-Panlab) and tap water *ad libitum*. One month after birth, the standard laboratory food was gradually replaced in all animals with the experimental diets (see below) at a rate of 25% per week; all mice were fed with 100% experimental diet from the age of 2 months until the time of sacrifice. For lipid analysis, a group of intact females fed with standard laboratory food was used as a control. La Laguna University Animal Care and Use Committee approved the protocol for this study.

### Ovariectomy and hormone treatments

In preliminary experiments it was found that ovariectomized mice implanted with 0.05 mg estradiol pellets (Innovative Research of America, Sarasota, Florida) for 90 days showed plasma estradiol levels of 7.2 ± 4.54 pg/ml (mean ± SEM) as determined by RIA (Architect System, ref #B7K720, Abbot, Germany). Since these values are within the range of those found in cycling animals of the same age at proestrus (20.9 ± 6.03 pg/ml) and estrus (1.8 ± 4.54 pg/ml), this dose of estradiol was chosen for the dietary experiments. Thus, female mice were anesthetized with inhaled isoflurane (2 ± 0.5%) after analgesic injection (buprenorphine hydrochloride, Buprex), and then bilaterally ovariectomized or sham-operated at 90 ± 1 days-of-age through a 1 cm dorsal incision that was closed with surgical clips. The day after ovariectomy mice received a 3 mm pellet containing a 90-day timed-release 0.05 mg 17β-estradiol or placebo. The pellet was subcutaneously implanted in the subscapular region by using a trocar according to manufacturer's instructions. Animals still showing inflammation around the implantation area 13 days after receiving the pellet were discarded. Mice were then housed according to the different diets and hormonal treatments until the end-point 90 days later, as shown in Table 1.

**Table 1:** Design of experimental groups.

**Reproductive status**	**Diet**
Intact sham-operated females (CO)	Standard laboratory food (SF)
	High *n*−6/*n*−3 ratio diet –(DI)
	Low *n*−6/*n*−3 ratio diet– (DII)
Ovariectomized-placebo treated (OVX)	High *n*−6/*n*−3 ratio diet–(DI)
	Low *n*−6/*n*−3 ratio diet– (DII)
Ovariectomized-estradiol treated (OVX-E)	High *n*−6/*n*−3 ratio diet– (DI)
	Low *n*−6/*n*−3 ratio diet– (DII)

### Diets

Two specific experimental diets were used in these experiments. The high *n*−6/*n*−3 ratio diet (DI) containing safflower oil, which had undetectable levels of EPA (20:5*n*−3) and DHA (22:6*n*−3), had abundant oleic acid (18:1*n*−9) and linoleic acid (18:2*n*−6), but was poor in α-linolenic acid (18:3*n*−3). The low *n*−6/*n*−3 ratio diet (DII) had the same basic composition, but was supplemented with extra EPA and DHA in a proportion of 7 g/kg of diet, added in the form of fish oil as a lipid source to give a particularly high DHA content. These diets were designed in the Instituto de Nutrición y Tecnología de los Alimentos at the University of Granada, Spain, and produced by Mucedola (Mucedola srl, Milano, Italy). Both diets and the standard laboratory food (SF) were subsequently lab-analyzed to determine the final percentage and absolute quantity (g/kg) of each FA (Table 2). Summarizing the main diet differences: (a) DI had a *n*−6/*n*−3 ratio 13–14 times higher than that of SF and 59 times higher than that of DII; (b) DII had a *n*−6/*n*−3 ratio 4–5 times lower than that of SF; (c) DI did not have traces of either DHA or EPA, while DII contained relevant amounts of both LC-PUFAs (Table 2). For simplicity, high and low *n*−6/*n*−3 LC-PUFA ratio diets are referred to in the text and graphics as DI and DII, respectively. Mice were fed these diets *ad libitum* for 90 days until the time of sacrifice.

**Table 2:** Main fatty acid composition of experimental diets (Mean ± *SD*, g/kg fresh weight).

**Fatty acids**	**SF**	**DI**	**DII**
C 14: 0	0.05 ± 0.00	0.21 ± 0.01	1.50 ± 0.00
C 16: 0	4.13 ± 0.14	4.68 ± 0.31	6.33 ± 0.04
C 16:1 *n*−7	0.09 ± 0.01	0.17 ± 0.01	1.55 ± 0.01
C 18:0	0.75 ± 0.04	1.55 ± 0.08	1.77 ± 0.01
C 18:1 *n*−9	13.15 ± 0.35	12.04 ± 0.81	6.15 ± 0.05
C 18:1 *n*−7	0.34 ± 0.06	0.59 ± 0.07	0.98 ± 0.02
C 18:2 *n*−6	10.63 ± 0.78	28.84 ± 0.42	16.23 ± 0.00
C 18:3 *n*−3	1.01 ± 0.13	0.15 ± 0.03	0.44 ± 0.00
C 18:4 *n*−3	0.00 ± 0.00	0.00 ± 0.00	0.66 ± 0.01
C 20:0	0.11 ± 0.01	0.18 ± 0.00	0.24 ± 0.00
C 20:1 *n*−9	0.18 ± 0.00	0.12 ± 0.00	1.09 ± 0.07
**C 20:4** ***n***−**6 (ADA)**	0.00 ± 0.00	0.00 ± 0.00	0.20 ± 0.00
C 20:4 *n*−3	0.00 ± 0.00	0.00 ± 0.00	0.24 ± 0.01
**C 20:5** ***n***−**3 (EPA)**	0.00 ± 0.00	0.00 ± 0.00	2.29 ± 0.02
C 22:0	0.18 ± 0.01	0.10 ± 0.01	0.13 ± 0.02
C 22:1 *n*−11	0.05 ± 0.02	0.10 ± 0.06	0.74 ± 0.04
**C 22: 5** ***n***−**6 (DPA)**	0.00 ± 0.00	0.00 ± 0.00	0.11 ± 0.01
C 22: 5 *n*−3	0.05 ± 0.00	0.06 ± 0.03	0.43 ± 0.01
**C 22: 6** ***n***−**3 (DHA)**	0.00 ± 0.00	0.00 ± 0.00	3.47 ± 0.01
**TOTALS**			
Saturates	5.42 ± 0.16	6.83 ± 0.37	10.41 ± 0.06
Monoenes	14.00 ± 0.39	13.41 ± 1.03	11.24 ± 0.09
PUFAs	11.69 ± 0.91	29.05 ± 0.47	24.07 ± 0.08
*n*−9	13.47 ± 0.33	12.35 ± 0.86	7.85 ± 0.10
*n*−6	10.63 ± 0.78	28.84 ± 0.42	16.55 ± 0.09
*n*−3	1.06 ± 0.13	0.22 ± 0.06	7.52 ± 0.01
*n*−3 LC-PUFAs	0.05 ± 0.00	0.06 ± 0.03	6.43 ± 0.02
*n*−6 LC-PUFAs	0.00 ± 0.00	0.00 ± 0.00	0.32 ± 0.01
**RATIOS**			
*n*−3/*n*−6	0.10 ± 0.01	0.01 ± 0.00	0.45 ± 0.00
*n*−6/*n*−3	10.04 ± 0.52	138.37 ± 33.71	2.20 ± 0.02
Total FA content	31.53 ± 1.67	49.84 ± 2.10	46.43 ± 0.45
% Lipids (fresh weight)	5.48 ± 0.11	6.45 ± 0.55	6.31 ± 0.00
% Moisture	9.79 ± 0.09	8.65 ± 0.27	7.85 ± 0.00
% Lipids (dry weight)	6.07 ± 0.12	7.06 ± 0.59	6.85 ± 0.00

*FA; fatty acids. Totals include some minor components not shown. The most common LC-PUFAs mentioned in the text are bold marked*.

### Tissue processing and sample preparation

Mice were sacrificed using CO_2_ 90 days after ovariectomy or sham operation, and their brains were collected. Cerebral cortex tissue for western blot analysis was homogenized in 3 volumes of ice-cold lysis buffer containing 20 mM HEPES, 100 mM NaCl, 100 mM NaF, 1 mM Na_3_VO_4_, 5 mM EDTA, 1% Triton X100 plus protease inhibitor cocktail (Roche Diagnostic) and 1 μM Okadaic acid (Calbiochem). Homogenates were centrifuged at 16,000 × g for 20 min at 4°C, and supernatants were stored at −80°C. Protein concentration was measured using BioRad DC Protein Assay (BioRad) following the manufacturer's protocol. Buffer containing 10% sodium dodecyl sulfate (SDS), 0.5 mM dithiothreitol, 325 mM Tris HCl, pH 6.8, 87% glycerol and bromophenol blue was added to samples before loading into polyacrylamide gels for electrophoresis. For all samples Western blot determinations were repeated 3–4 times. For lipid analysis, tissues were homogenized at a concentration of 5 mg/ml in PBS with 0.01% 3,5-Di-tert-4-butylhydroxytoluene (HBT) as an antioxidant.

### Western blot analysis

Cerebral cortex tissue extracts were resolved by SDS-PAGE and transferred onto nitrocellulose (Whatman) or PVDF (Millipore) membranes, and subsequently blocked by incubation in 10% non-fat milk for 1 h at room temperature. Membranes were incubated overnight at 4°C with appropriate primary antibodies (Table 3), then washed in 0.1% Tween–PBS and incubated with secondary horseradish peroxide-conjugated antibody (Santa Cruz Biotechnology, Santa Cruz, CA, USA). Antibody binding was detected with *SuperSignal*^*TM*^ (Thermo-Fisher Scientific), using β-actin as internal loading control. Densitometry analysis was performed using a GS-800 Calibrated Densitometer (Bio-Rad).

**Table 3:** Antibodies used in western blot analysis.

**Antibody**	**Source**	**Dilution**	**References**
P120	Mouse	1:1,000	#610133 BD Transduction Lab, USA
PSD95	Rabbit	1:1,000	#3450 Cell Signaling, USA
Phospho-Synapsin (Ser-9)	Rabbit	1:1,000	#2311 Cell Signaling, USA
Synapsin	Rabbit	1:1,000	#2312 Cell Signaling, USA
Synaptophysin (SY38)	Mouse	1:20,000	#10701 Progen, UK
β-catenin	Mouse	1:1,000	#610153 BD, USA
βAmyloid, 1-16 (6E10)	Mouse	1:1,000	#SIG-39320 Covance, USA
BACE (D10E5)	Rabbit	1:1,000	#5606 Cell Signaling, USA
Tau1	Mouse	1:1,000	#MAB3420 Chemicon, USA
Tau5	Mouse	1:1,000	#AHB0042 Invitrogen, USA
PHF1	Mouse	1:1,000	Dr. P. Davies, USA
GFAP	Rabbit	1:1,000	#G5601 Promega, USA
β-actin	Mouse	1:10,000	#A4441 Sigma-Aldrich, USA
Anti-mouse IgG-HRP	Goat	1:5,000	#sc-2005 St Cruz Biotech, Germany
Anti-rabbit IgG-HRP	Goat	1:5,000	#sc-2004 St Cruz Biotech, Germany

### Lipid analysis

Lipid extraction from dietary samples and cerebral tissue was performed using a modification of the Folch's method (Folch et al., 1957). Dietary FA profiles (g FA/kg diet fresh weight) were obtained by means of acid-catalyzed transmethylation of the lipid fractions followed by GC-MS analysis (Fabelo et al., 2014). Similarly, the cerebral cortex lipid fractions were subjected to further analysis of FA and complex lipids as previously described (Cingolani et al., 2014). Sphingolipids were analyzed by HPLC-MS (Garanto et al., 2013) by using 0.2 nmol of C17-sphinganine, *N*–dodecanoylsphingosine, *N*–dodecanoylglucosylsphingosine, and *N*–dodecanoyl sphingosylphosphorylcholine as internal standards. Sphingolipids were annotated as <lipid subclass> <total fatty acyl chain length>: <total number of unsaturated bonds>. If the sphingoid base residue was dihydrosphingosine, the lipid class contained a <DH> prefix. Since our chromatographic separation did not discriminate between dh-glucosylceramides (GlcdhCer) and dh-galactosylceramides (GaldhCer), their mixture was represented as monohesoxylceramides (MHC). In all cases, the final tissue data was indicated as pmol/mg of protein, except in the case of total FA, which were calculated as pmol equivalents *per* mg of protein with respect to C12 ceramide. For statistical analysis, all lipids were shown as percentage of variation relative to its content in brain tissue from animals fed with SF.

### Statistical analysis

For lipidomic outcomes, we performed a 2 × 3 factorial analysis with an extra control group. General ANOVA was performed, followed by linear contrasts to answer the questions posed during the design phase of the study. Regardless, no more than 6 contrasts were performed. In this way, 12 response variables were tested in addition to the “multi-testing effect,” whose accurate quantification is unfeasible. A reasonable approach to take this into account is to consider only those effects or interactions in which the *p*-value of the test is <0.01. However, when any interaction was found to be significant in several of the analyzed lipids, it is discussed, even if the *p-*value of the test is slightly higher than 0.01. In addition, the 95% confidence interval was calculated for all effects and interactions of interest.

In the case of changes in cerebral cortex protein expression levels, since the effect of diet under each reproductive status was monitored in different electrophoresis gels, it was not feasible to evaluate possible effects of each hormone condition. However, we were still able to evaluate the interaction of the hormonal condition with the effect of the diet. Thus, for each reproductive status and within the same gel, we calculated the percentage of variation induced by DII vs. DI diets. Then, in order to estimate effects and interactions between the different factors, we ran a saturated regression model which includes the dichotomous variable diet and the polytomous variable hormone condition with three levels (Armitage et al., 2004). The latter entered the model as two dummy variables, the first with a value of “1” in ovariectomized animals receiving placebo pellets, and the second with value “1” in ovariectomized mice treated with estradiol. Intact, sham-operated controls had a value of “0” in each dummy variable. This model provides us with confidence intervals and tests for the three effects of the diet conditioned to the hormonal status and the interaction between the two factors, adding up to the 5 comparisons “a priori” expected in the experimental design. Although there are four animals per group, the 2 × 3 factorial design includes 6 × 4 = 24 animals, which allows us to estimate the intragroup population variance with 6 × 3 = 18 degrees of freedom (df). In this way, any contrast that compares two means follows a t-Student distribution with 18 df. This provides a higher statistical power than the simple comparison of two groups with 4 animals per group.

For all tests, actual *p*-values are given for each comparison and the significance of the main effects detected by the analysis. The statistical software used was Stata 14 (StataCorp LLC, College Station, Texas) for inference factorial analysis and GraphPad Prisma 7 (GraphPad Software, Inc., San Diego, California) for graphic representations.

## Results

### The cerebral cortex lipidome is affected by dietary differences in the ratio of *n*−3 and *n*−6 LC-PUFAs

**Figure 1:**
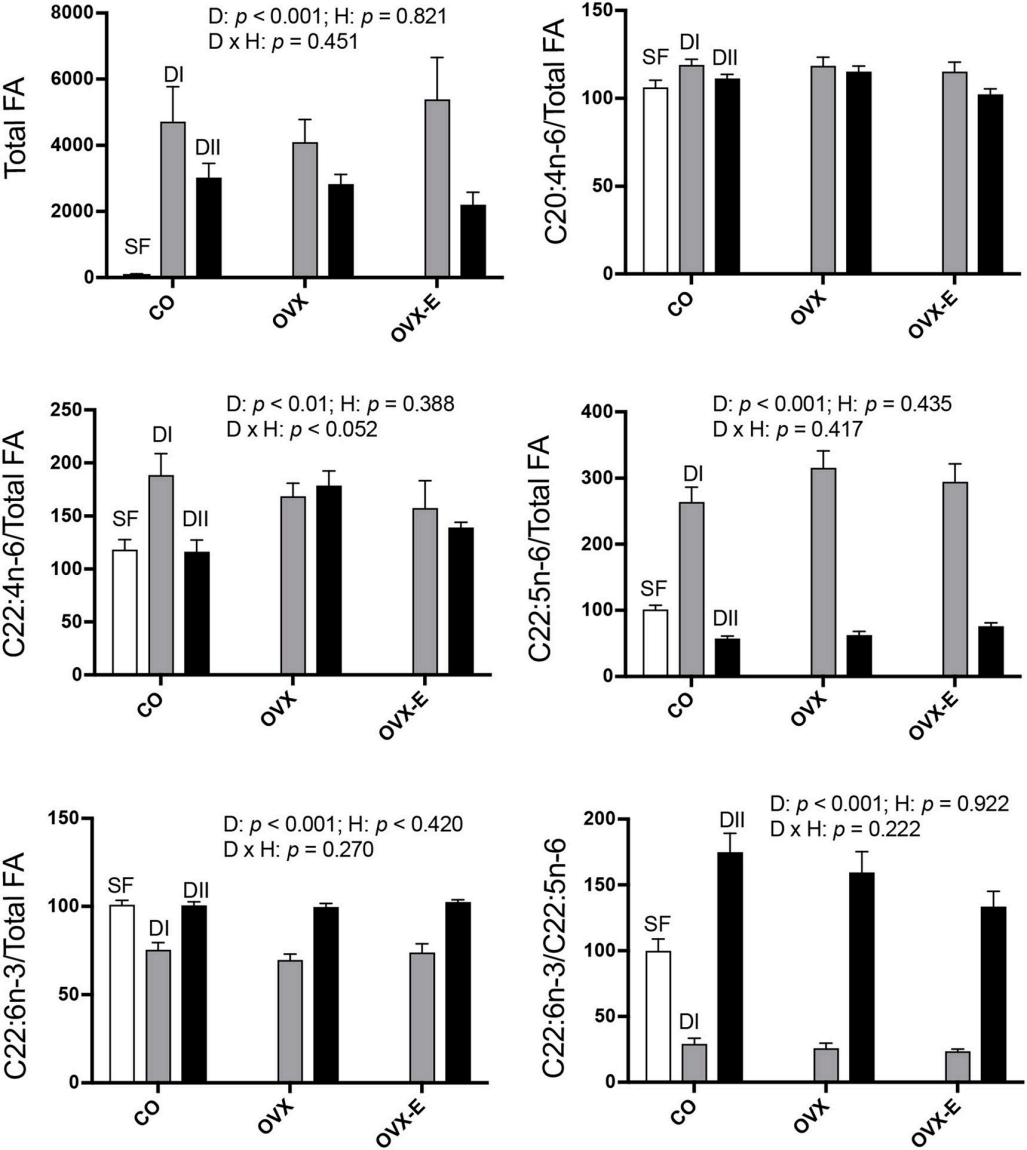
Differential effects of dietary LC-PUFAs on the relative cerebral cortex fatty acid content in female mice under different reproductive conditions. Fatty acid (FA) composition of each diet is shown in Table 1. The FAs from brain cortex were obtained and analyzed as described in section Materials and Methods. White bars represent the relative FA content from cerebral cortex of intact animals fed with standard food (SF). Gray and black bars represent the data from animals fed with DI or DII *n*−6/*n*−3 LC-PUFA diets, respectively. Vertical axis represents the percentage of variation induced by each diet relative to SF. Data are represented as mean ± SEM of 4 animals per group, except those fed with SF (*n* = 3). Horizontal axis indicates the different reproductive status: CO, intact, sham-operated; OVX, ovariectomized, placebo-treated; OVX-E, ovariectomized, estradiol-treated. Only some representative *p-*values for the main effects of diet (D), hormone status (H) and their interaction (D × H) were indicated in each graph and the remaining data were detailed in Table 4.

In intact, sham-operated females (CO), both experimental diets (DI and DII) induced a dramatic rise in total brain cortex FAs (Figure 1 and Table 4A), compared with animals fed with SF (DI: *p* = 0.0001; DII: *p* = 0.015) and the effect of DI was even higher than that of DII (*p* = 0.004). The effect of diet was not significantly affected by reproductive status (*p* = 0.821), and no significant interaction was detected (*p* = 0.451). Thus, cerebral cortex levels of total FAs were altered by changes in the ratios of dietary *n*−3 and *n*−6 LC-PUFAs, apparently regardless of the presence of ovaries and circulating levels of estradiol. With regard to the LC-PUFAs analyzed, each was significantly altered by both experimental diets compared to their levels in animals fed with SF (*p* < 0.01 or *p* < 0.001). In the case of C20:4*n*−6 (arachidonic acid, ARA) almost no modifications were seen (Figure 1 and Table 4B), in parallel with the dietary content of its precursor, C18:2*n*−6 (linoleic acid), which had the lowest levels in mice fed with SF, and the highest in those fed with DI (Table 2). In contrast, levels of C22:4*n*−6 (adrenic acid; ADA) and C22:5*n*−6 (DPA) were elevated in animals fed with DI (high *n*−6/*n*−3 ratio) when compared to mice fed with SF or DII (with lower *n*−6/*n*−3 ratio), as shown in Figure 1, Table 4C (*p* = 0.0091) and Table 4D (*p* < 0.0001). Regarding the levels of C22:6*n*−3 (DHA), they were reduced in animals fed with DI (high *n*−6/*n*−3 ratio) compared to mice fed with either SF or DII (low *n*−6/*n*−3 ratio), as shown in Figure 1 and Table 4E (*p* < 0.0001). However, no significant differences were found between the levels of DHA in animals fed with SF and those fed with DII, in agreement with the high amount of its precursor, C18:3*n*−3 (α-linolenic acid) in both. Despite its high concentration in diet DII, we were unable to detect any trace of 20:5*n*−3 (EPA), the most direct DHA biosynthetic precursor. Interestingly, the ratio between C22:6*n*−3 (DHA) and C22:5*n*−6 (DPA) was found to be significantly reduced in animals fed with DI and elevated in those fed with DII as shown in Figure 1 and Table 4F (*p* < 0.0001, and *p* = 0.0001), in correlation with their higher *n*−6/*n*−3 and lowest *n*−6/*n*−3 ratios, respectively.

**Table 4:** Linear contrasts of the differential effects of dietary *n*−6/*n*−3 ratio on cerebral cortex fatty acid content under different reproductive conditions.

**Lipid class**	**Main effects**	***p-*value**	**95% CI**
A. Total FAs	DI-SF (CO)	**0.0001**	**2618.3, 6650.0**
	DII-SF (CO)	**0.0154**	**558.5, 4631.7**
	DI-DII (CO)	**0.0041**	**730.7, 3350.4**
B. C20:4*n*−6	DI-SF (CO)	0.0288	1.3, 12.4
	DII-SF (CO)	0.4942	−6.8, 13.6
	DI-DII (CO)	0.0194	1.4, 14.5
C. C22:4*n*−6	DI-SF (CO)	**0.0091**	**19.7, 121.2**
	[DI-DII (CO)] - [DI-DII (OVX)]	**0.0194**	**16.1, 148.9**
D. C33:5*n*−6	DI-SF (CO)	**>0.00001**	**142.7, 237.8**
	DII-SF (CO)	0.1375	−83.7, 12.5
	DI-DII (CO)	**>0.00001**	**195.0, 256.8**
E. C22:6*n*−3	DI-DII (CO+OVX+OVX–E)	**>0.00001**	−**33.4**, −**22.4**
F. C22:6*n*−3/C22:5*n*−6	[SF (CO)] - [DI (CO+OVX+OVX–E)]	**>0.00001**	**47.8, 99.8**
	[DII (CO)] - [SF (CO)]	**0.0001**	**29.7, 82.3**

*DI: High dietary n−6/n−3 ratio; DII: Low n−6/n−3 ratio; SF: Standard food. CO: Intact control female mice; OVX: Ovariectomized, placebo-treated female mice; OVX–E: Ovariectomized, estradiol-treated female mice. Data represented in Figure 1 were analyzed by 2 × 3 factorial ANOVA. In cases where the influence of reproductive status on diet action was absent and significant interaction between the factors was not detected, data from those animals were grouped to emphasize the main dietary effects. The most significant p-values and 95% confidence intervals are highlighted*.

In addition to the effect of dietary composition on brain FA content, we studied its possible dependence on reproductive status. As shown in Figure 1, no clear effect was found in either ovariectomized or estradiol-treated mice, and only in the case of C22:4*n*−6 (ADA) was a nearly significant interaction detected (*p* = 0.052). In addition, when comparing the differential effect of DI and DII in control (CO) vs. ovariectomized (OVX) mice, a truly significant difference was found (Table 4C: *p* = 0.0194). Since the effect of diet was quite similar under all reproductive conditions for the rest of FAs analyzed, we grouped the data from CO, OVX, and OVX-E mice to emphasize the effect and ratio of DI and DII in the case of C22:5*n*−6 (DPA) and C22:6*n*−3 (DHA) (Tables 4E,F). It was found that, irrespective of reproductive condition, the effect of each diet on cortical levels of these LC-PUFAs correlated comparatively more with the total amount of FAs or relatively with the amount of linoleic acid in the diet (Table 2).

**Figure 2:**
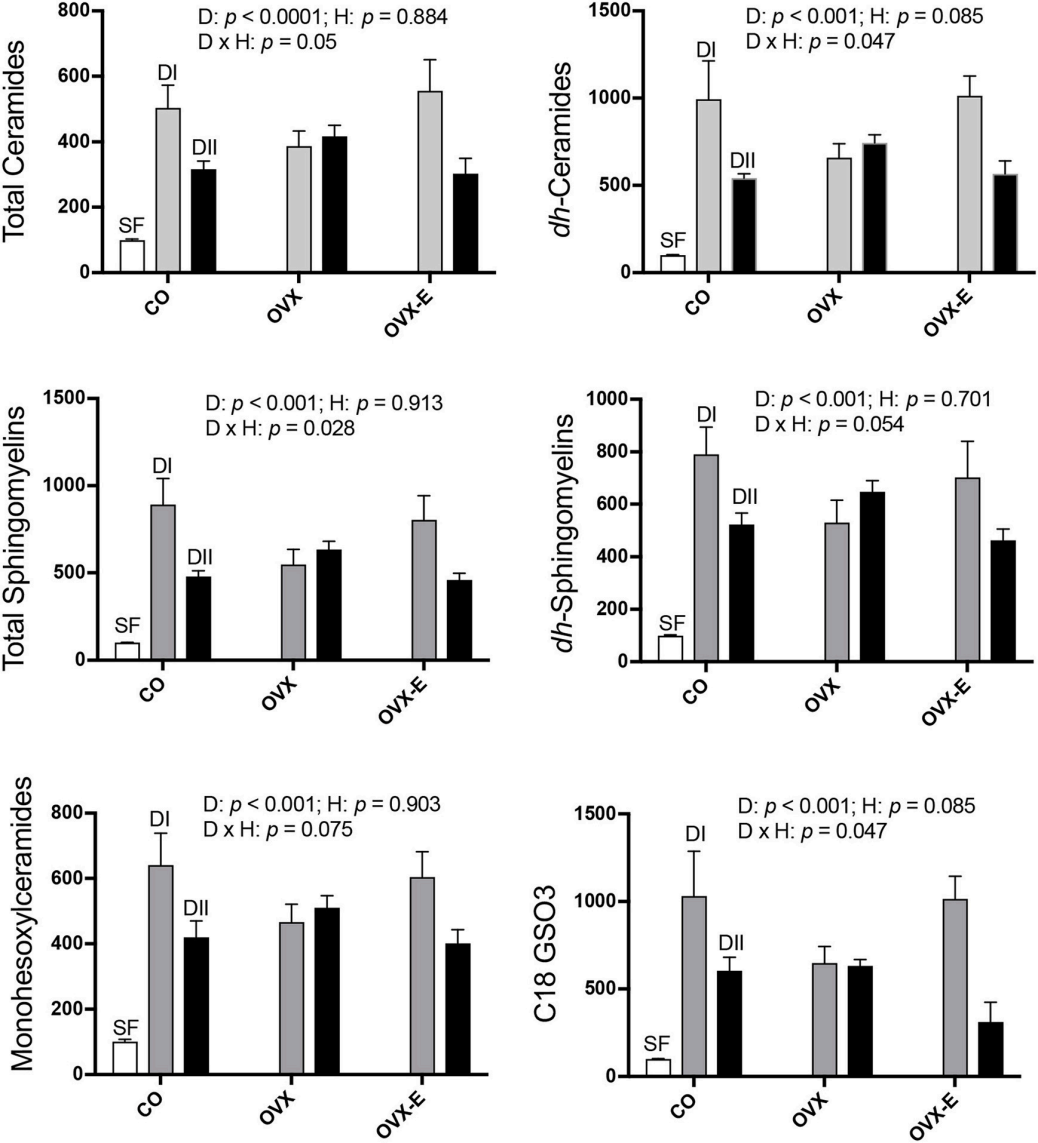
Differential effects of dietary LC-PUFAs on the relative complex lipid content in the cerebral cortex of female mice under different reproductive conditions. Some sphingolipids and complex lipids were obtained and analyzed as described in section Materials and Methods. White bars represent the relative lipid content from cerebral cortex of intact animals fed with standard laboratory food (SF). Gray and black bars represent the same data from animals fed with DI or DII *n*−6/*n*−3 LC-PUFA diets, respectively. Vertical axis represents the percentage of variation induced by each diet relative to SF. Data are represented as mean ± SEM of 4 animals per group, except for those fed with SF (*n* = 3). Horizontal axis indicates the different reproductive status: CO, intact, sham-operated; OVX, ovariectomized, placebo-treated; OVX-E, ovariectomized, estradiol-treated The data were analyzed as previously described, and only some representative *p-*values for the main effects of diet (D), hormone status (H) and their interaction (D × H) were indicated in each graph, the remaining data were detailed in Table 5.

Results from the analysis of complex lipids are shown in Figure 2 and Table 5; for all lipids analyzed, the global effect of diet was highly significant (*p* ≤ 0.001–0.0001). Thus, control animals fed alternative diets had increased cerebral cortex complex lipids compared to those fed SF, although the effect was significantly, quantitatively higher in mice fed with DI (*p* ≤ 0.0001) than those fed with DII (*p* ≤ 0.02–0.003). On the other hand, though no significant effect of hormone status was observed, certain interactions between diet and reproductive condition (D × H) were detected (Figure 2), a finding that justified further comparisons of the differences between the effects of both diets under each reproductive condition (Table 5; *p-*values and 95% confidence intervals). In all cases, the differences between the DI and DII effects observed in control animals were also significant in ovariectomized, estradiol-treated mice, and were even more evident when data from CO and OVX-E groups were combined. In contrast, the effects of DI and DII were similar in ovariectomized, placebo-treated females and no significant differences were detected for any of the different lipids analyzed. Furthermore, when comparing the dietary effect observed in CO animals (intact ovaries) plus OVX-E animals (continuous estradiol treatment) with that of OVX mice, a reasonable level of significance was found in all cases (Tables 5A–F, *p* ≤ 0.025).

**Table 5:** Linear contrasts of the differential effects of dietary *n*−6/*n*−3 ratio on cerebral cortex lipid content under different reproductive conditions.

**Lipid class**	**Main effects**	***p-*value**	**95% CI**
A. Total Ceramides	DI-SF (CO)	**0.0001**	**230.1, 578.3**
	DII-SF (CO)	**0.018**	**41.3, 389.5**
	DI-DII (CO)	**0.024**	**27.6, 350.0**
	DI-DII (OVX)	0.707	−190.6, 13.8
	DI-DII (OVX–E)	**0.007**	**78.8, 427.1**
	DI-DII (CO+OVX–E)	**0.001**	**102.2, 339.5**
	[DI-DII (CO+OVX–E)]-[DI-DII (OVX)]	**0.017**	**50.1, 450.4**
B. *dh-*Ceramides	DI-SF (CO)	**>0.00001**	**551.9, 1236.4**
	DII-SF (CO)	**0.014**	**99.3, 783.8**
	DI-DII (CO)	**0.008**	**135.7, 769.5**
	DI-DII (OVX)	0.577	−402.9, 230.8
	DI-DII (OVX–E)	**0.013**	**104.6, 789.1**
	DI-DII (CO+OVX–E)	**0.001**	**216.5, 682.9**
	[DI-DII (CO+OVX–E)]-[DI-DII (OVX)]	**0.010**	**142.3, 929.2**
C. Total Sphingomyelins	DI-SF (CO)	**>0.00001**	**500.9, 1082.9**
	DII-SF (CO)	**0.013**	**88.0, 669.9**
	DI-DII (CO)	**0.005**	**143.5, 682.3**
	DI-DII (OVX)	0.512	−355.5, 183.3
	DI-DII (OVX–E)	**0.023**	**54.4, 636.4**
	DI-DII (CO+OVX–E)	**0.001**	**180.9, 577.4**
	[DI-DII (CO+OVX–E)] - [DI-DII (OVX)]	**0.009**	**130.7, 799.7**
E. MHC	DI-SF (CO)	**>0.00001**	**432.0, 948.6**
	DII-SF (CO)	**0.003**	**165.7, 682.3**
	DI-DII (CO)	**0.031**	**27.2, 505.4**
	DI-DII (OVX)	0.323	−355.1, 123.2
	DI-DII (OVX–E)	0.067	−18.9, 497.7
	DI-DII (CO+OVX–E)	**0.007**	**76.8, 428.8**
	[DI-DII (CO+OVX–E)] - [DI-DII (OVX)]	**0.018**	**71.8, 665.7**
E. MHC	DI-SF (CO)	**>0.00001**	**346.9, 734.7**
	DII-SF (CO)	**0.003**	**126.8, 514.6**
	DI-DII (CO)	**0.019**	**40.6, 399.6**
	DI-DII (OVX)	0.589	−226.6, 132.4
	DI-DII (OVX–E)	**0.041**	**8.8, 396.6**
	DI-DII (CO+OVX–E)	**0.003**	**79.3, 343.5**
	[DI-DII (CO+OVX–E)] - [DI-DII (OVX)]	**0.025**	**35.6, 481.4**
F. C18 GSO3	DI-SF (CO)	**0.0001**	**519.4, 1343.0**
	DII-SF (CO)	**0.019**	**93.1, 916.6**
	DI-DII (CO)	**0.030**	**45.1, 807.6**
	DI-DII (OVX)	0.934	−366.0, 396.5
	DI-DII (OVX–E)	**0.001**	**321.0, 1083.5**
	DI-DII (CO+OVX–E)	**0.0001**	**294.7, 833.9**
	[DI-DII (CO+OVX–E)] - [DI-DII (OVX)]	**0.024**	**82.1, 1016.0**

*DI: High dietary n−6/n−3 ratio; DII: Low n−6/n−3 ratio; SF: Standard food. CO: Intact control female mice; OVX: Ovariectomized, placebo-treated female mice; OVX–E: Ovariectomized, estradiol-treated female mice. Data represented in Figure 2 were analyzed by 2 × 3 factorial ANOVA. In cases where the influence of reproductive status on diet action was absent and significant interaction between the factors was not detected, data from those animals were grouped to emphasize the main dietary effects. The most significant p-values and 95% confidence intervals are highlighted*.

As an indication of the levels of estradiol, the dry uterine weight was determined at the endpoint. Intact females showed similar uterine weights, with independence of being fed with DI or DII (DI: 16.00 ± 2.40 mg; DII: 18.40 ± 2.00 mg). Also, OVX animals treated with estradiol showed similar uterine weights with the two diets (DI: 38.40 ± 8.00 mg; DII: 34.40 ± 3.20 mg). In contrast, OVX mice treated with placebo and fed with DI had uterine weights significantly higher than those fed with DII (DI: 8.00 ± 1.20 mg; DII: 4.00 ± 0.40 mg; *p* < 0.001).

In summary, these findings suggest interaction of diet and ovarian hormones on the levels of complex lipids in the mouse cerebral cortex, even though the presence or absence of ovaries and normal circulating estradiol levels do not appear to significantly modify the main effect of each diet.

### The expression of synaptic proteins in the cerebral cortex are affected by dietary differences in the ratio of *n*−3 and *n*−6 LC-PUFAs

Brain lipids constitute a basic component of neural organization and play a critical role in the interactions of membrane proteins that are involved in cell signaling and synaptic functions (Bazan, 2014), processes that are also regulated by circulating levels of reproductive hormones and locally-synthesized steroids (Varea et al., 2009; Arevalo et al., 2015). Thus, it was interesting to explore whether differences in dietary *n*−6/*n*−3 LC-PUFA ratios affect expression and/or phosphorylation of synaptic proteins and key neuronal markers. Statistical analyses of observed changes for each protein were performed as follows: (a) the effect of differences in dietary content of LC-PUFAs in control animals; (b) the global interaction between the effect of diet and reproductive status; and (c) the specific differences between the effect of diet in controls and those observed in OVX or OVX-E mice.

**Figure 3:**
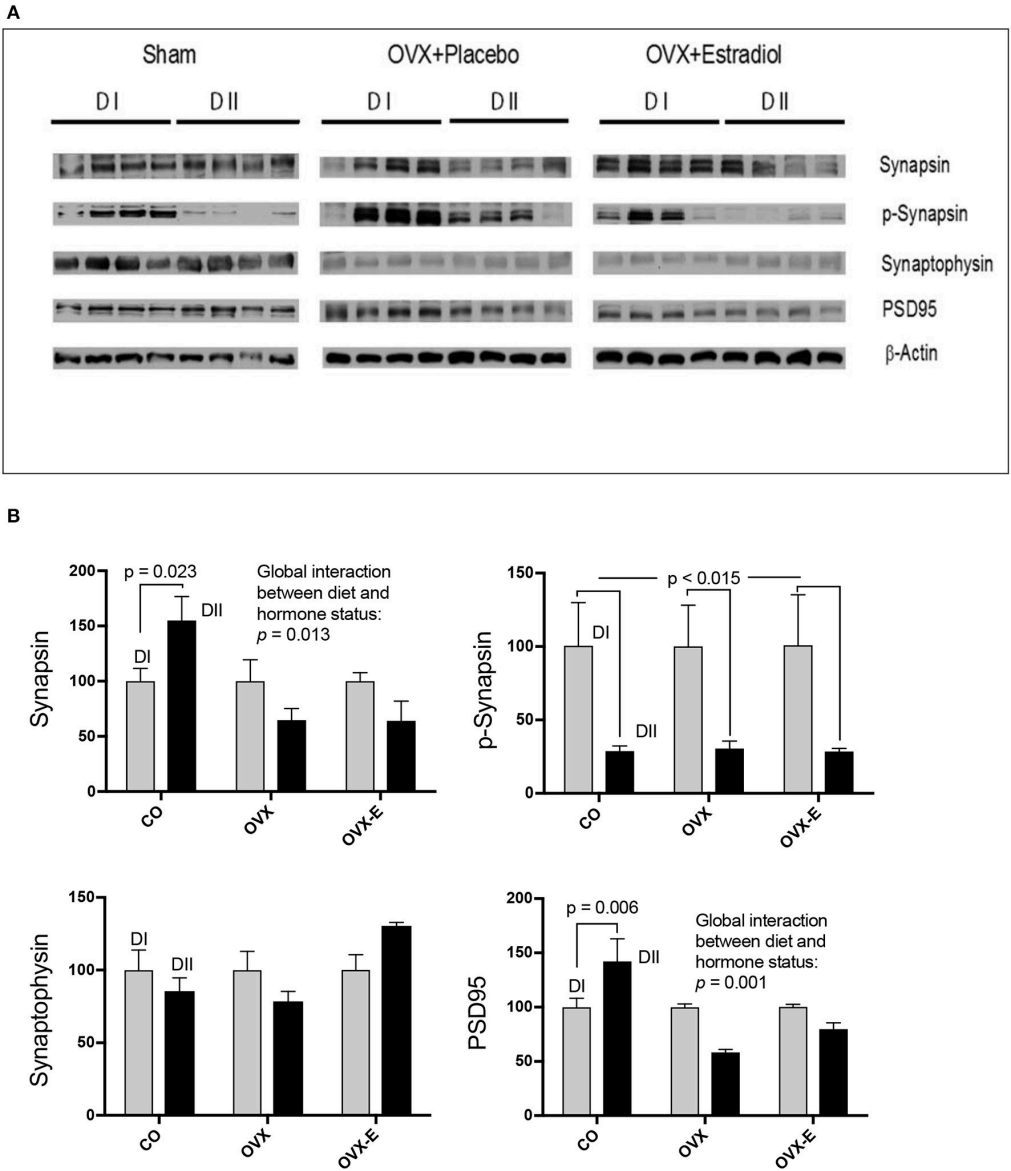
Differential effects of dietary LC-PUFAs on the expression of synaptic proteins (synapsin, synaptophysin, and PSD95) in the cerebral cortex of female mice under different reproductive conditions. **(A)** Represents the Western blots, and **(B)** represents means ± SEM of densitometric quantification (4 mice per group). Western blots were quantified and normalized with respect to the loading control, actin. The normalized data corresponding to intact, sham-operated mice from the DI diet was arbitrarily considered 100 relative units. Vertical axis represents the percentage of variation induced by DII vs. DI *n*−6/*n*−3 LC-PUFA ratio diet. Horizontal axis indicates the different reproductive status as follows: CO, intact, sham-operated; OVX, ovariectomized, placebo-treated; OVX-E, ovariectomized, estradiol-treated. The data were statistically analyzed as previously described, and for simplicity, only the main effects of diet are given in the figure, as well as the interaction between diet and reproductive status. Other details of the analysis including the differences between the effect of diet in controls and ovariectomized mice treated with either placebo or estradiol were presented in Table 6.

**Figure 4:**
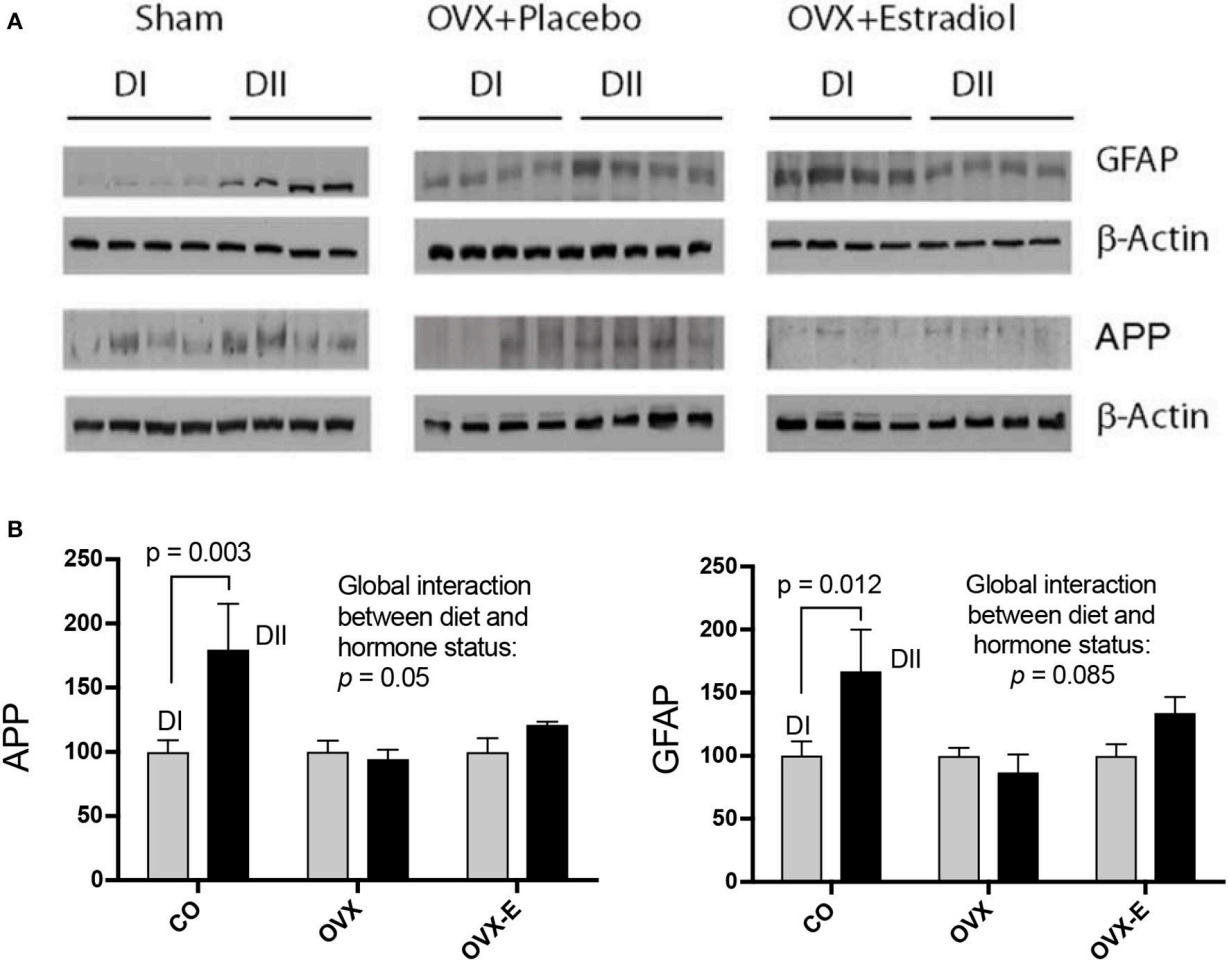
Differential effects of dietary LC-PUFAs on the expression of APP and GFAP in the cerebral cortex of female mice under different reproductive conditions. **(A)** Represent the western blots, and **(B)** represent the densitometric data as means ± SEM of relative densitometric quantification (4 mice per group). Vertical axis represents the percentage of variation induced by DII vs. DI *n*−6/*n*−3 LC-PUFA ratio diet. Horizontal axis indicates the different reproductive status as follows: CO, intact, sham-operated; OVX, ovariectomized, placebo-treated; OVX-E, ovariectomized, estradiol-treated. Western blots were quantified and normalized with respect to the loading control, β-Actin. The normalized data corresponding to intact, sham-operated mice from the DI diet was arbitrarily considered 100 relative units. The data were statistically analyzed as previously described, and only some relevant data are included in this Figure whereas the remaining data was presented in Table 7.

CO mice fed a low *n*−6/*n*−3 ratio diet (DII) showed higher levels of both synapsin (*p* = 0.023) and PSD-95 (*p* = 0.006) than those fed with a high *n*−6/*n*−3 ratio diet (DI) (Figure 3 and Tables 6A, D). In both cases, a global interaction between diet and reproductive status was observed (*p* = 0.013 and *p* = 0.001, respectively). Significant differences were also found in the effect of diet on the levels of synapsin (Table 6A: *p* = 0.001 and *p* = 0.009) and PSD-95 (Table 6D: *p* = 0.0004 and *p* = 0.004) when intact (CO) mice were compared with ovariectomized (OVX) or ovariectomized, estradiol-treated animals (OVX-E), respectively. These findings suggest that the presence of ovaries may be necessary for the effect of dietary lipid composition on the expression of these synaptic proteins, and that continuous treatment with estradiol alone was not able to compensate for ovariectomy. In contrast, levels of p-synapsin were reduced in animals fed with a low *n*−6/*n*−3 ratio diet (DII) compared to those fed with a high *n*−6/*n*−3 ratio diet (DI), regardless of reproductive status (Figure 3 and Table 6B: *p* = 0.015). These differences in p-synapsin were additionally confirmed by a supplementary Western blots from the three diets (DI, DII and SF), and using PDK1 and Actin as internal controls (Supplementary Figure 1). No significant effects were found in the case of synaptophysin (Figure 3 and Table 6C), P120 or β-catenin (Tables 6E,F, respectively). The slight effect observed in synaptophysin in the case of OVX-E animals fed DII when compared to CO mice was not considered significant as it was quantitatively small, and the 95% confidence intervals did not allow further accurate interpretation of the results. Neither significant effects of diet nor reproductive status were found in the case of several neuronal markers such as BACE, PHF1, Tau1, and Tau5 (data not shown). However, CO mice fed with low *n*−6/*n*−3 ratio diet (DII) showed higher levels of APP and GFAP than those fed with high *n*−6/*n*−3 ratio diet (DI) (Figure 4 and Table 7; *p* = 0.003 and *p* < 0.0012, respectively). These effects were partially affected by reproductive status, as certain global interaction was detected, albeit of a small magnitude. Significant differences were also detected in the effect of diet on both APP and GFAP between controls and ovariectomized animals (Tables 7A,B; *p* = 0.018 and *p* = 0.029, respectively), which was partially compensated by continuous estradiol treatment.

**Table 6:** Linear contrasts of the differential effects of dietary *n*−6/*n*−3 ratio on the expression of synaptic proteins in the cerebral cortex of female mice under different reproductive conditions.

**Protein**	**Main effects**	***p-*value**	**95 % CI**
A. synapsin	DI-DII (CO)	**0.023**	**8.5, 101.4**
	[DI-DII (CO)] - [D-DII (OVX)]	**0.001**	−**155.7**, −**24.4**
	[DI-DII (CO)] - [DI-DII (OVX–E)]	**0.009**	−**156.4**, −**25.1**
B. p-synapsin	DI-DII (CO)	**0.014**	−**191.9**, −**25.3**
	[DI-DII (CO)] - [DI-DII (OVX)]	0.988	−117.0, 118.7
	[DI-DII (CO)] - [DI-DII (OVX–E)]	0.478	−77.2, 158.5
C. synaptophysin	DI-DII (CO)	0.321	−44.3, 15.4
	[DI-DII (CO)] - [DI-DII (OVX)]	0.730	−49.2, 35.1
	[DI-DII (CO)] - [DI-DII (OVX–E)]	**0.039**	**2.5, 86.9**
D. PSD-95	DI-DII (CO)	**0.006**	**13.8, 70.8**
	[DI-DII (CO)] - [DI-DII (OVX)]	**0.0004**	−**124.3**, −**43.6**
	[DI-DII (CO)] - [DI-DII (OVX–E)]	**0.004**	−**103.0**, −**22.3**
E. P120	DI-DII (CO)	0.440	−33.8, 74.4
	[DI-DII (CO)] - [DI-DII (OVX)]	0.293	−115.9, 37.1
	[DII-DII (CO)] - [DI-DII (OVX–E)]	0.830	−84.4, 68.6
F. β-catenin	DI-DII (CO)	0.217	−82.2, 20.0
	[DI-DII (CO)] - [DI-DII (OVX)]	0.142	−19.5, 125.0
	[DI-DII (CO)] - [DI-DII (OVX–E)]	0.122	−16.4, 128.1

*The effect of diet under each reproductive status was calculated as a percentage of variation induced by DII (Low n−6/n−3 ratio) vs. DI (High dietary n−6/n−3 ratio). CO: Intact, sham-operated female mice; OVX: Ovariectomized, placebo-treated; OVX-E: Ovariectomized, estradiol-treated. Data represented in Figure 3 were analyzed by 2 × 3 factorial ANOVA and saturated regression models; the most significant p values and their corresponding 95% confidence intervals are highlighted*.

**Table 7:** Linear contrasts of the differential effects of dietary *n*−6/*n*−3 ratio on the expression of APP and the glial marker GFAP, in the cerebral cortex of female mice under different reproductive conditions.

**Protein**	**Main effects**	***p-*value**	**95 % CI**
A. APP	DI-DII (CO)	**0.003**	**31.1, 128.3**
	DI-DII (CO) - DI-II (OVX)	**0.018**	−**154.2**, −**16.7**
	DI-DII (CO) - DI-DII (OVX–E)	0.090	−127.4, 10.1
B. GFAP	DI-DII (CO)	**0.012**	**16.7, 117.0**
	DI-DII (CO) - DI-II (OVX)	**0.029**	−**150.7**, −**9.0**
	DI-DII (CO) - DI-DII (OVX–E)	0.344	−103.7, 38.1

*The effect of diet under each reproductive status was calculated as percentage of variation induced by DII (Low n−6/n−3 ratio) vs. DI (High dietary n−6/n−3 ratio). CO: Intact, sham-operated female mice; OVX: Ovariectomized, placebo-treated; OVX-E: Ovariectomized, estradiol-treated. Data represented in Figure 4 were analyzed by 2 × 3 factorial ANOVA and saturated regression model; the most significant p-values and their corresponding 95% confidence intervals are highlighted*.

## Discussion

Experimental evidence has suggested that the ratio between *n*−6 and *n*−3 LC-PUFAs is a critical factor for their developmental and neuroprotective activity (Miller et al., 2016; Dyall, 2017). Therefore, the present study tested the effects of two experimental diets, DI and DII, whose major difference in composition was the global ratio of *n*−6 and *n*−3 LC-PUFAs. In addition, the two diets contained higher total levels of FA content (DI: 158%; DII: 147%) compared to the standard diet (SF), which explains the dramatic increases of total FAs observed in the cerebral cortex of mice fed with both experimental diets.

As expected, DI had increased percentages of *n*−6 FAs including C22:4*n*−6 (ADA) and C22:5*n*−6 (DPA), and reduced levels of C22:6*n*−3 (DHA). In addition, both diets caused minor elevation in C20:4*n*−6 (ARA) levels in the cerebral cortex, though the effect of DI was slightly higher, in agreement with the dietary content of its precursor, linoleic acid. Since ARA is a critical intermediate in the biosynthesis of thromboxanes, prostaglandins, and leukotrienes, which play an essential role in the inflammatory/anti-inflammatory response (Stables and Gilroy, 2011), some regulatory mechanisms to maintain balanced levels may exist. On the other hand, levels of DHA were higher in the cortex of mice fed with DII than in those fed with DI, in good correlation with the absence of DHA and the extremely low levels of its precursor, C18:3*n*−3 (ALA) in the composition of DI. Interestingly, the levels of DHA in animals fed with DII were similar to those fed with SF, despite the absence of DHA in the standard diet. However, the fact that the SF diet contains twice as much of the DHA precursor indicates that ALA-derived DHA may be sufficient to maintain brain DHA levels and preserve its function, as it has been suggested (Anderson et al., 2005; André et al., 2005). Furthermore, this interpretation is in agreement with evidence from animal models that brain DHA levels are similar when fed with diets with ALA as the only PUFA compared to those fed with DHA or ALA+DHA (reviewed in Barcelo-Coblijn and Murphy, 2009). Regarding other LC-PUFA intermediates in DHA biosynthesis, such as eicosatetranoic acid (ETA, C20:4*n*−3) or EPA (C20:5*n*−3), we did not find detectable amounts of either in brain samples, in agreement with previous reports (Miller et al., 2016; Harauma et al., 2017).

In summary, the most relevant finding regarding the effect of DI and DII on LC-PUFAs was a dramatic reduction in the ratio between C22:6*n*−3 and C22:5*n*−6 in animals fed with DI, and a marked increase in those fed with DII. However, this effect does not seem to be due to DHA enrichment in DII, but rather to the differential dietary levels of LNA (DI) and ALA (DII), in agreement with other reports (Domenichiello et al., 2016). Interestingly, no significant effects of reproductive status were detected for the dietary effects on cerebral cortex LC-PUFA levels, since the *p-*values obtained from the factorial analysis did not support relevant interactions between diet and gonadal conditions.

Our findings also show that the two experimental diets induced profound and highly significant elevations in the levels of all complex lipids analyzed, though for all of them the effect was stronger in animals fed a diet with the highest *n*−6/*n*−3 LC-PUFA ratio. Interestingly, the effects of DI and DII on cortical ceramide levels observed in control mice were not detected in ovariectomized mice. Furthermore, the differential effect in all complex lipids of each diet was reverted in ovariectomized animals receiving continuous estradiol treatment. These results suggest a synergistic action between dietary PUFAs and ovarian hormones on the cerebral cortex lipidome, which would be in agreement with other reports using hippocampal tissue from female mice and multivariate statistical approaches (Díaz et al., 2016).

The influence of ovarian hormones on the dietary effects on cortical ceramides is highly relevant given the important function of these lipids in a wide range of cell processes including growth, differentiation, apoptosis, and oncogenesis (Kashara and Sanai, 2000; Simons and Toomre, 2000; Anderson and Jacobson, 2002; Sengupta et al., 2007; Pruett et al., 2008). Increase in ceramide concentration in cell membranes affects not only the structural organization and dynamic properties of lipid rafts (Cremesti et al., 2002) but also myelin formation and stability (Pan et al., 2005; Susuki et al., 2007), neural differentiation (Wang and Yu, 2013; Wang et al., 2014), synapse formation (Hering et al., 2003; Mendez-Otero and Santiago, 2003), synaptic plasticity and transmission, neurotoxicity, and neurodegeneration (Hering et al., 2003; Besshoh et al., 2005; Ferrer, 2009; Fabelo et al., 2014; Attiori Essis et al., 2015; Sonnino and Prinetti, 2016; Marín et al., 2017). In addition to experimental evidence in rodents, a number of postmortem studies support the role of age-dependent or genetic alterations of ceramide and sphingolipid metabolism in several neurological disorders, including Alzheimer's (AD) and Parkinson's diseases (He et al., 2010; Fabelo et al., 2011; Gegg et al., 2012; Bouti et al., 2016; Olsen and Færgeman, 2017). However, despite the importance of ceramide and sphingolipids for brain function, the effect of dietary interventions on their levels in the cerebral cortex has not been previously explored.

In addition, neuronal lipid microdomains are also targets for estrogen hormones that act via classical estrogen receptors or membrane-associated receptors to trigger a variety of signaling pathways (Marín et al., 2003, 2005, 2007; Guerra et al., 2004). Activation of these pathways is involved in the regulation of brain development, neuronal survival and synaptic plasticity, which are the basis for the neuroprotective role of estrogens (Marín et al., 2005; Herrera et al., 2011; Arevalo et al., 2015). Therefore, dietary and estrogen-induced modification of brain sphingolipid composition may be expected to alter the expression of specific signaling and synaptic proteins. Thus, the second aim of this work was to determine whether circulating ovarian hormones could interact with the effects of dietary lipid composition, altering expression of neuronal proteins in the mouse cerebral cortex. We analyzed the levels of key neuronal proteins such as P120, PSD-95, β-catenin, synaptophysin, and synapsin. Though no significant effects were detected on the expression of P120, β-catenin, or synaptophysin, both PSD-95 and synapsin were found to be sensitive to the different dietary *n*−6/*n*−3 ratios. In addition, the effect of diet was blocked by ovariectomy, suggesting a potential synergy between diet and reproductive hormones at both presynaptic and postsynaptic levels.

Synapsins are a family of presynaptic proteins that interact with synaptic vesicles through phosphorylation-dependent processes (Chi et al., 2003; Cousin et al., 2003; Menegon et al., 2006; Sun et al., 2006; Giachello et al., 2010; Messa et al., 2010). In this work, a diet with a low *n*−6/*n*−3 LC-PUFA ratio (with relatively high levels of DHA and EPA) induced higher expression of synapsin, but lower levels of p-synapsin (Ser-9) than a diet with a higher *n*−6/*n*−3 ratio (lacking supplemental DHA and EPA). Interestingly, regarding total synapsin, it was found that ovariectomy blocked the dietary effect, and that continuous estradiol-treatment neither reverted nor prevented the effect of ovariectomy, suggesting that, in addition to estradiol, other ovarian secretions might be involved. In this respect, estrogen-progesterone interactions in the modulation of synaptic plasticity and the expression of both pre-and post-synaptic proteins in several brain regions of monkeys and rodents have been reported (Choi et al., 2003; Foy et al., 2008; Williams et al., 2011; Baudry et al., 2013). On the contrary, the profound dietary effect observed for p-synapsin was not influenced by ovariectomy. It should be pointed out that synapsin phosphorylation is a short-term regulated event at the level of presynaptic terminals which depends on action potential firing, activation dynamics of highly localized voltage-dependent calcium channels, and a complex interplay between several Ca^++^-dependent protein kinases (Chi et al., 2003; Menegon et al., 2006; Sun et al., 2006; Giachello et al., 2010). Thus, protein-lipid interactions at the level of presynaptic membranes can be expected to depend on dietary lipid composition and being additionally influenced by ovarian function. However, rapid modulation of short-term presynaptic phenomena, such as those involved in p-synapsin phosphorylation and its potential dependence on ovarian hormone levels, must be difficult to be observed under our experimental approach.

PSD-95 is an integral component of the postsynaptic density that participates in neurotransmission (Cho et al., 1992; Kim and Sheng, 2004; Berry and Nedivi, 2017), synaptic plasticity and learning (Migaude et al., 1998; EI-Husseini et al., 2000; Schnell et al., 2002; Ehrlich and Malonow, 2004; Ehrlich et al., 2007). In intact female mice, we found that a diet with a low *n*−6/*n*−3 LC-PUFA ratio (enriched in DHA and EPA) induced higher expression levels of PSD-95 in the cerebral cortex than a diet with a higher *n*−6/*n*−3 ratio (lacking DHA or EPA enrichment). This is in agreement with previous reports of reduced PSD-95 expression in aged mice fed with DHA-deficient diets (Sidhu et al., 2016). This effect was not observed in ovariectomized animals, indicating that circulating ovarian hormones might interact with brain dietary inputs for regulation of the synaptic proteome. However, administration of estradiol alone was unable to either revert or prevent the effect of ovariectomy. Even though we cannot reach clear conclusions from these findings, they suggest that, in addition to estradiol, oscillations in other cyclic ovarian secretions may play a physiological role in the modulation of brain cortex synaptic proteins by dietary differences in *n*−6/*n*−3 ratios. In this respect, as mentioned above, there are several reports supporting the potential effect of estrogen-progesterone interactions on synaptic plasticity and post-synaptic proteins (Foy et al., 2008; Baudry et al., 2013; for review see Williams et al., 2011).

The second group of proteins analyzed were those involved in some neurodegenerative pathologies like AD and other taupathies, such as APP, Tau, BACE or GFAP (for review see Laird et al., 2005; LaFerla and Green, 2012; Rosenberg et al., 2016). Our data indicate that only APP and GFAP were differentially expressed in the cerebral cortex of female mice fed with either DI or DII. In both cases, ovariectomy blocked the differential effect of DII, even though only a slight estradiol-induced reversion was observed. It is tantalizing to suggest that the increase of APP expression induced by DII over DI might be a positive response to certain stress, influencing the balance of neuronal-glia interactions. It will be interesting to further studying the effects of these dietary and reproductive manipulations in specific mouse models of neurodegenerative disorders.

The results of the current work indicate that different dietary *n*−6/*n*−3 LC-PUFA ratios are able to remodel the lipidome in the cerebral cortex of female mice, and that these effects are partially influenced by the circulating levels of ovarian hormones. A variety of results from animal models of neurodegenerative diseases have suggested that dietary interventions may be useful therapeutic tools against several neurodegenerative disorders (Lim et al., 2005; Green et al., 2007; Bazan et al., 2011a,b; Bazan, 2014). However, the potential for these therapeutic approaches in human health and disease is still unclear, and the current dietary recommendations have been considered specific to particular age groups and physiological conditions (Zárate et al., 2017). The interactions between the effect of dietary lipid composition and reproductive status shown here indicate the importance the specific *n*−6/*n*−3 composition in therapeutic diets, which must also be well-balanced in regards to the hormonal context and physiological situations of age-associated impairment of reproductive function, such as menopause.

## Author contributions

JH performed the animal experiments and was in charge of most experimental manipulations, hormone treatments, and collection of brain samples; JH and LO-G prepared brain samples and western blotting analysis; AM and GH participated in experimental designs, animal treatments, and collection of samples; GF and JC performed the lipidomic analysis of brain samples; NA and CR analyzed the lipid composition of the experimental diets; CR collaborated in the interpretation of results; LP-V performed statistical analysis of quantitative results; RA conceived and designed the experiments and contributed together with LP-V to the design of the specific statistical analysis; LG-S, RA, and FW were in charge of the final interpretation of results and wrote the paper.

### Conflict of interest statement

The authors declare that the research was conducted in the absence of any commercial or financial relationships that could be construed as a potential conflict of interest.
