# Supplementary material for: Ovarian Function Modulates the Effects of Long-Chain Polyunsaturated Fatty Acids on the Mouse Cerebral Cortex
Source: Front Cell Neurosci. 2018 Apr 24;12:103. doi: 10.3389/fncel.2018.00103 (PMC5928148; doi:10.3389/fncel.2018.00103)
Supplement: Supplementary file 1 [file Image_1.pdf]

Supplementary Figure 1

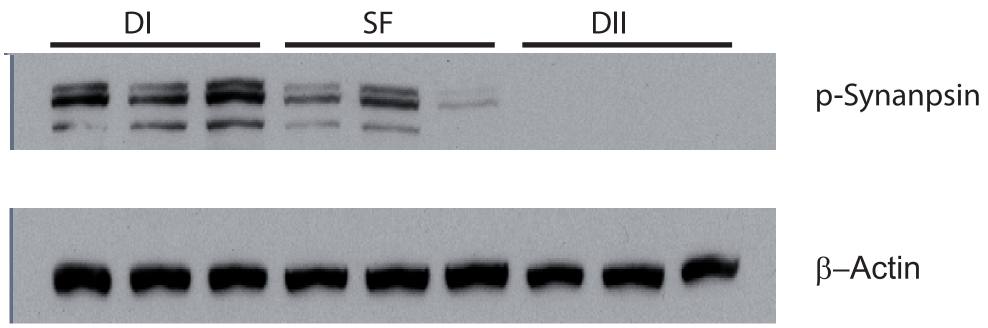

Western blots (WBs) from three arbitrary samples from sham-mice group , fed with DI, DII or SF.
WBs (25 ug/lane) were analysed with antibodies against pSynapsin (as presented in the manuscritp, Figure 3) and β-actin as loading control

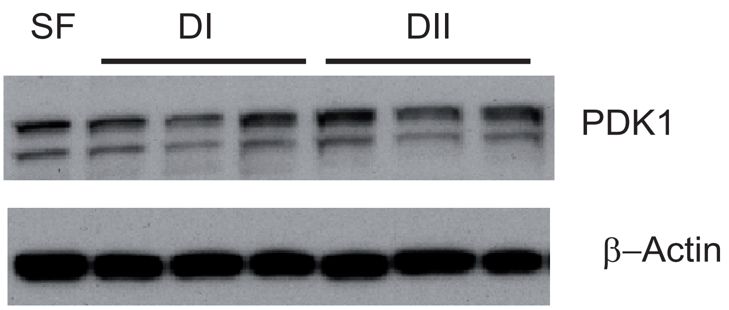

Western blos from three arbitrary samples from sham-mice group fed with DI, DII and one sample from SF. WBs (25 ug/lane) were analysed with antibodies against PDK1 and β-actin. Not appreciable differences were observed in both cases.
